# Green Tea Polyphenols and Padma Hepaten Inhibit* Candida albicans* Biofilm Formation

**DOI:** 10.1155/2018/1690747

**Published:** 2018-09-30

**Authors:** Yosi Farkash, Mark Feldman, Isaac Ginsburg, Doron Steinberg, Miriam Shalish

**Affiliations:** ^1^Biofilm Research Laboratory, Institute of Dental Sciences, Faculty of Dentistry, Hebrew University-Hadassah, P.O. Box 12065, Jerusalem 91120, Israel; ^2^Department of Orthodontics, Hebrew University-Hadassah School of Dental Medicine, P.O. Box 12272, Jerusalem 91120, Israel; ^3^Microbiology Research Laboratory, Institute of Dental Sciences, Faculty of Dentistry, Hebrew University-Hadassah, P.O. Box 12065, Jerusalem 91120, Israel

## Abstract

*Candida albicans (C. albicans)* is the most prevalent opportunistic human pathogenic fungus and can cause mucosal membrane infections and invade the blood. In the oral cavity, it can ferment dietary sugars, produce organic acids and therefore has a role in caries development. In this study, we examined whether the polyphenol rich extractions Polyphenon from green tea (PPFGT) and Padma Hepaten (PH) can inhibit the caries-inducing properties of* C. albicans*. Biofilms of* C. albicans* were grown in the presence of PPFGT and PH. Formation of biofilms was tested spectrophotometrically after crystal violet staining. Exopolysaccharides (EPS) secretion was quantified using confocal scanning laser microscopy (CSLM). Treated* C. albicans* morphology was demonstrated using scanning electron microscopy (SEM). Expression of virulence-related genes was tested using qRT-PCR. Development of biofilm was also tested on an orthodontic surface (Essix) to assess biofilm inhibition ability on such appliances. Both PPFGT and PH dose-dependently inhibited biofilm formation, with no inhibition on planktonic growth. The strongest inhibition was obtained using the combination of the substances. Crystal violet staining showed a significant reduction of 45% in biofilm formation using a concentration of 2.5mg/ml PPFGT and 0.16mg/ml PH. A concentration of 1.25 mg/ml PPFGT and 0.16 mg/ml PH inhibited candidal growth by 88% and EPS secretion by 74% according to CSLM. A reduction in biofilm formation and in the transition from yeast to hyphal morphotype was observed using SEM. A strong reduction was found in the expression of* hwp1, eap1,* and* als3* virulence associated genes. These results demonstrate the inhibitory effect of natural PPFGT polyphenolic extraction on* C. albicans* biofilm formation and EPS secretion, alone and together with PH. In an era of increased drug resistance, the use of phytomedicine to constrain biofilm development, without killing host cells, may pave the way to a novel therapeutic concept, especially in children as orthodontic patients.

## 1. Introduction

*C. albicans* is the most prevalent opportunistic human pathogenic fungus, which can cause infections of mucosal membranes (candidiasis) and invade the blood stream (candidemia) [1]. It is able to form biofilms on mucosal membranes as well as on implants [2]. Biofilm formation and virulence of* C. albicans* are related to its transition from the yeast to the hyphae morphotype, which represents a crucial step towards pathogenesis.

Hyphae provide structural integrity to biofilms [3].* C. albicans* has been found in periodontal pockets in both the chronic and aggressive forms of periodontitis [4].

*C. albicans* is a common colonizer of carious lesions in children and in adolescents. It can ferment and/or assimilate some dietary sugars and produce organic acids in the dental plaque and therefore has a role in caries development [5]. An in vitro study revealed that the occurrence of caries in children was positively correlated with the frequency of oral candidal carriage [6].

*C. albicans* virulence is associated with the expression of extracellular polysaccharides (EPS), such as glucans and mannans. These EPS act as adhesins, which enable the adhesion of* C. albicans* to host epithelial cells. They are also required for host recognition [7].

Hyphal wall protein 1 (HWP1) is a well-characterized* C. albicans* cell-surface protein, required for hyphal development and yeast adhesion to epithelial cells. HWP1 is known to be required for* C. albicans* biofilm formation in vivo and thus may be an excellent therapeutic target [8].

EAP1 gene, which encodes a glycosylphosphatidylinositol-anchored glucan-cross-linked cell wall protein, and agglutinin-like sequence 3 (ALS3), which encodes a cell-surface glycoprotein, also have a role in adhesion and biofilm formation [9, 10].

Previous studies have shown that oral appliances increase the oral* Candida* carriage rate and cause significant population alterations [11, 12]. Moreover, it was found that the* Candida* counts increase during orthodontic treatment with fixed appliances [13–16].

*C. albicans* and additional catalase-rich microbiota may have the ability to scavenge oxidants and thus can protect catalase-negative anaerobes and facultative anaerobes cariogenic streptococci against peroxide and thus secure their survival in the oral cavity.

This oxidative scavenging ability is markedly potentiated by chlorhexidine [17].

Polyphenols are abundant micronutrients in our diet, which endow their beneficial effect as antioxidants mainly in the oral cavity, due to saliva which enhances their solubilization [18] and downstream also in the stomach [19].

Green tea, extracted from* Camellia sinensis*, is a widely consumed beverage throughout the world, second only to water [20]. Green tea contains multiple polyphenolic catechin components, and epigallocatechin-3-gallate (EGCG) is the primary catechin accounting for 50–80% in a brewed cup [21]. Epicatechin-3-gallate (ECG) is the second most concentrated catechin component of green tea and is associated with its anti-inflammatory/antioxidant properties [22]. Other major catechins found in green tea include epicatechin (EC) and epigallocatechin (EGC).

Padma Hepaten is a polyphenolic formula derived from traditional Tibetan medicine, produced by Padma Inc. (Schwerzenbach, Switzerland). In Tibetan it is called “three fruits” and is composed of Chebulic myrobalan, amla fruit, and belleric myrobalan in the ratio 2:1:1 [23]. It was shown that Hepatic fibrosis is significantly ameliorated by PH administration [24]

Since polyphenols were previously shown to inhibit* C. albicans* biofilm formation [25, 26], the goal of this study was to examine whether PPFGT and PH have an inhibitory effect on* C. albicans* biofilm formation and can participate in the prevention of candidiasis, candidemia, and caries in the general population and in orthodontic patients.

## 2. Materials and Methods

### 2.1. Materials

Polyphenon 60 from green tea was purchased from Sigma- Aldrich (St. Louis, MO, USA) and Padma Hepaten was obtained from Padma Inc. (Schwerzenbach, Switzerland). Both powders were dissolved and diluted in brain-heart infusion (BHI) to different concentrations.

### 2.2. Biofilm Growth

*C. albicans* SC5314 cells, kept in a glycerol stock at −80°C, were thawed and incubated on BHI agar plates for 18 h at 37°C. The cells were then diluted in BHI broth to an optical density (OD) of 0.05 at 595 nm using the Tecan GENios machine (Tecan US, Durham, NC) in 96-well microtiter plates. 2% sucrose was added to each well. The growth medium was supplemented with different concentrations of the polyphenol rich PPFGT and PH.* C. albicans* without PPFGT and PH served as a control. The biofilms were allowed to develop for 48 h, at 5% CO_2_ at 37°C.

### 2.3. Optical Density of Grown Biofilms after Crystal Violet Staining

The biofilm was stained with crystal violet (CV) for 30 minutes, then the CV was washed and the biofilm was later washed three times with phosphate buffered saline (PBS). The remaining color was extracted using 33% acetic acid. Then, the OD was measured spectrophotometrically at 595 nm using the Tecan GENios machine (Tecan US, Durham, NC) [27].

### 2.4. Confocal Laser Scanning Microscopy (CLSM)

In order to quantify the biomass of* C. albicans* and its EPS, confocal laser scanning microscopy was used. The biofilm was prepared as described above but instead of wild type* C. albicans* SC5314, we used* C. albicans* SC5314 carrying the GFP reporter gene (*C. albicans*–GFP), kindly provided by J. Berman (Tel Aviv University, Israel).

Forty-eight-hour biofilms developed in the presence of PPFGT (1.25 mg/ml) and PH (0.16 mg/ml) at the minimal concentration, which inhibited the biofilm growth by 50% (MBIC_50_), were washed with PBS and incubated for 45 minutes with concanavalin A-Alexa Fluor 647 conjugate (Con A; 25 mg/ml) (Invitrogen, Carsbad, CA, USA). Con A (excitation wavelength 650 nm and emission at 668 nm) selectively binds to the glucose and mannose residues of fungal cell wall EPS [28]. Stained EPS and microorganisms were observed by a Zeiss LSM 510 CLS microscope (Carl Zeiss, Oberkochen, Germany). Three-dimensional images of the fungus and EPS distribution within the biofilm were constructed using Zen2009 software (Carl Zeiss, New York, USA). At least three random fields were observed and analyzed. The number of the cells, as well as individual EPS production by* C. albicans,* were calculated as a color-appropriated fluorescence intensity, using ImageJ v3.91 software (http://rsb.info.nih.gov/ij). The data is presented as the amount of cells as well as of the individual EPS production by* C. albicans* cells in each layer of the biofilm (10 *μ*m). The percentage of total EPS production and total biomass in the biofilm formed in the presence of PH was calculated as the area under the curve (AUC) and compared to placebo control.

### 2.5. Morphology of the Biofilm

The assay was performed as described previously [29]. After washing, the biofilm was fixed in 4% formaldehyde for 1 hour at room temperature. The morphology of the cells in the biofilm formed in the presence of PPFGT was then visualized using an analytical Quanta 200 Environmental High-Resolution Scanning Electron Microscope (FEI, Eindhoven, Netherlands) at 200X–10,000X magnification. At least three random fields were observed and analyzed.

In this experiment the biofilm was grown on polyvinyl chloride (PVC) orthodontic surfaces (Essix) to check whether biofilm inhibition can also occur on orthodontic surfaces. Prior to biofilm formation, the PVC was disinfected by immersion in ethanol 70% overnight.

### 2.6. Quantitative Real-Time RT-PCR Analysis of* C. albicans* Specific Genes in the Biofilm

The assay was performed similarly to that described previously [29]. The biofilm was grown in the absence and presence of 1.25 mg/ml PPFGT and 0.16 mg/ml PH in 6-well plates under the conditions described above. After washing with PBS, biofilm cells were removed from the bottom of the plates with a sterile scraper and disrupted in a FastPrep Cell Disrupter (Bio101, Savant Instruments, Inc., NY, USA). Total RNA was extracted from the biofilm using Tri-Reagent (Sigma-Aldrich). RNA concentration was determined spectrophotometrically using a Nanodrop ND-1000 Instrument (Wilmington, DE, USA). Two micrograms of template was reverse-transcribed with Super Script First Strand (Invitrogen, Life Technologies, Carlsbad, CA, USA). The integrity and purity of the RNA were assessed using an Agilent 2100 Bioanalyzer system (Agilent Technologies, Santa Clara, CA, USA). Expression of* C. albicans* biofilm formation and stress associated genes (*hwp1, eap1, and als3*) was analyzed. The relative expression levels of the target genes were analyzed using an ABI-Prism 7300 Instrument (Applied Biosystems, Foster City, CA, USA). Platinum SYBR Green PCR Master Mix (Invitrogen) was used to monitor the amplified product in real time, following the manufacturer's protocol. Primers for the tested genes are listed in Supplementary Table S1. Three independent experiments were performed. To normalize the results, we used the expression of 18s rRNA.

### 2.7. Statistical Analysis

The statistical analysis was performed using Student's one tailed t-test (equal variances assumed) with a significance level of P < 0.05.

## 3. Results

**Figure 1:**
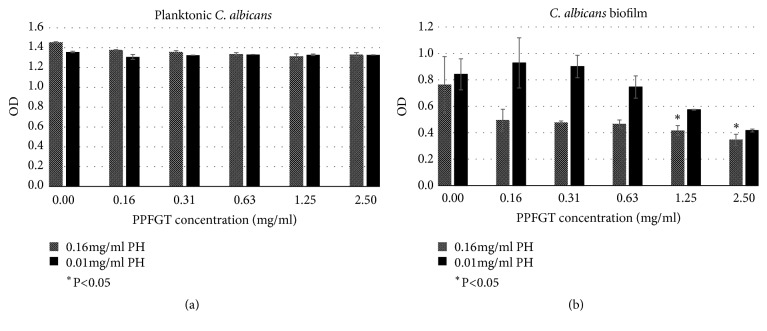
**Total biomass of planktonic and biofilm cells of* C. albicans *stained with crystal violet.** (a) OD of planktonic* C. Albicans* with increasing concentrations of PPFGT. (b) OD of biofilm of* C. albicans* with increasing concentrations of PPFGT. Error bars indicate the standard deviation (n=96).

PPFGT inhibited* C. albicans* biofilm growth (Figure 1). While there was a dose-dependent inhibition on biofilm cells, there was no such an effect on planktonic cells. Concentrations of 1.25 and 2.5 mg/ml PPFGT significantly inhibited biofilm formation. Those same concentrations did not inhibit planktonic growth. The addition of higher concentration of PH (0.16 mg/ml instead of 0.01 mg/ml) resulted in a stronger inhibition on the biofilm formation, suggesting a combined effect between the two substances.

**Figure 2:**
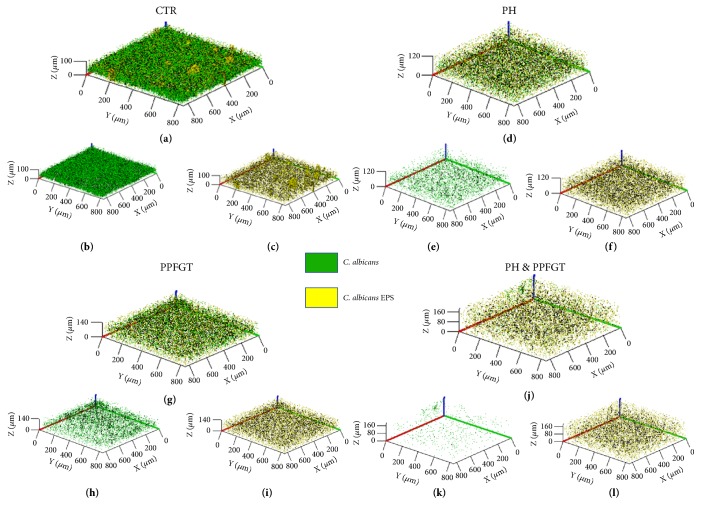
**Biofilm 3D reconstruction of confocal laser scanning microscopy.** Computerized 3D reconstruction (X, Y, and Z axis) of the biofilm layers (in *µ*m), recorded using CLSM and generated by the zen2009 software. (a) Control candida cells and EPS. (b) Candida cells only. (c) EPS only. (d) PH treated biofilm, both Candida cells and EPS. (e) Candida cells only. (f) EPS only. (g) PPFGT treated biofilm, both Candida cells and EPS. (h) Candida cells only. (i) EPS only. (j) PH and PPFGT treated biofilm, both Candida cells and EPS. (k) Candida cells only. (l) EPS only.

The 3D image of the reconstructed biofilm layers (Figures 2(a)-2(l)) visualizes the strong inhibition that PPFGT, PH, and their combination have on biofilm formation and EPS secretion by* C. albicans.* The treated biofilm consisted of less* candida* cells and less EPS and that the use of the two polyphenols together yielded the strongest inhibition.

**Figure 3:**
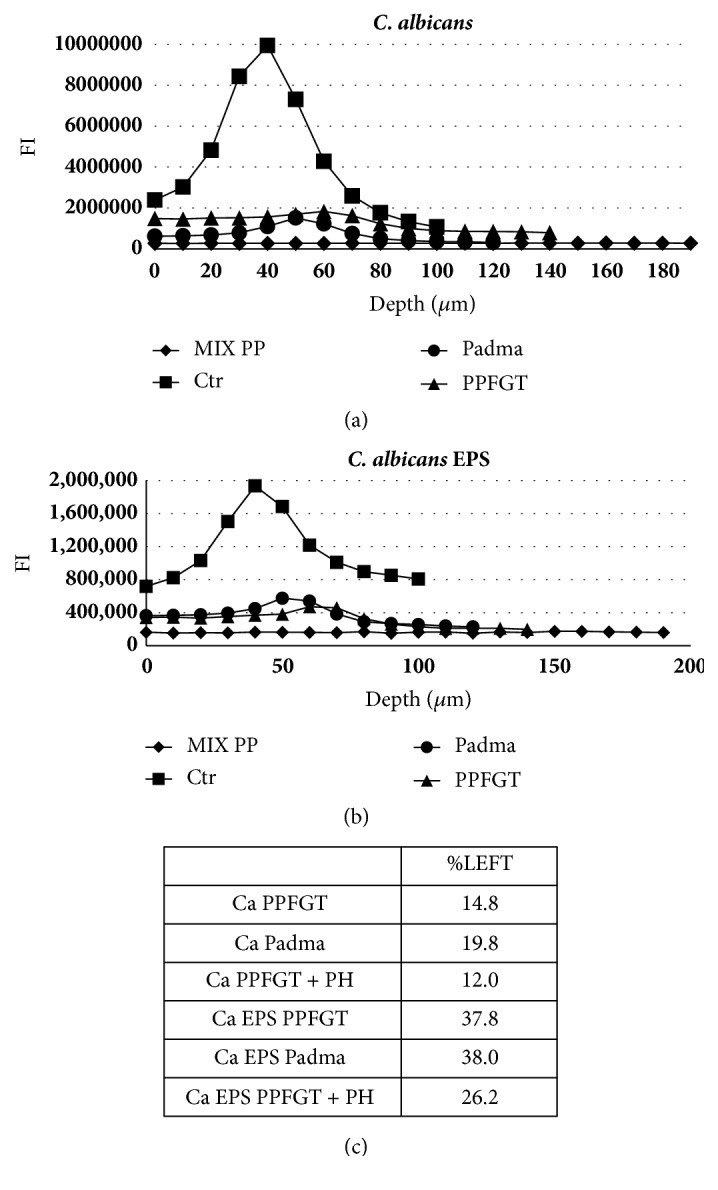
**Quantification of the confocal laser scanning microscopy results.** Charts (a) + (b) show the fluorescence intensity (FI) in each layer of the biofilm depth. (a) Cells quantification of the control and the treated groups. (b) EPS quantification of the control and the treated groups. (c) A table summing up the percentage of cells and EPS left after the various treatments relative to the control.

Figures 3(a)-3(c) provide a numerical analysis of these results. The bell-shaped charts characterize the* Candida* and EPS counts in the different layers of the biofilm. The highest counts are in the middle layers of the biofilm. The number of* Candida* cells and the EPS was reduced dramatically by the treatment with PPFGT and PH, and most significantly when they were combined. The cells growth was inhibited by 88% and the EPS production by 74% when the polyphenol mix was used at concentrations of 0.16 mg/ml PH and 1.25 mg/ml PPFGT.

**Figure 4:**
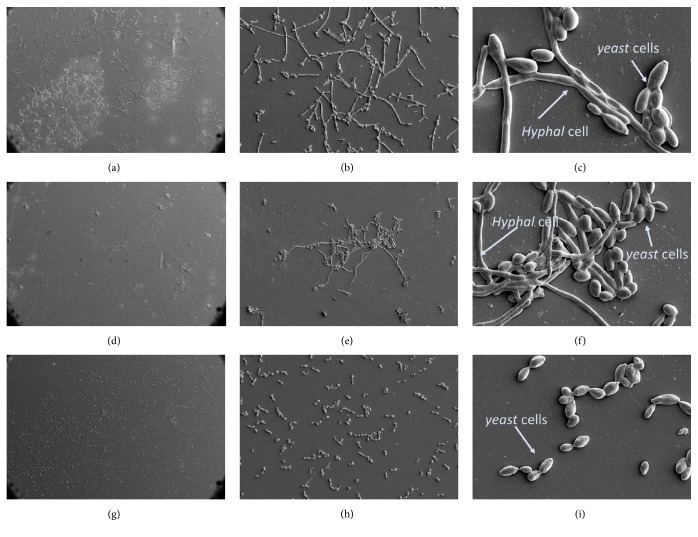
**Biofilm morphology on orthodontic PVC using SEM.** Morphology of the biofilm using SEM. (a) Control group at X200 magnification, (b) at X1,000, and (c) at X5,000. (d) PPFGT treated* C. albicans* at X200 magnification, (e) at X1,000, and (f) at X5,000. (g) PPFGT + PH treated at X200 magnification, (h) at X1,000, and (i) at X5,000.

The SEM results (Figure 4) indicated a reduction in cell number in the PPFGT group (Figures 4(d)-4(f)) in comparison to the control group (Figures 4(a)-4(c)). Figures 4(g)-4(i) show the morphology of* C. albicans* treated with both polyphenols. The treated group contains more yeast shaped cells and less hyphal cells, suggesting that PPFGT with PH may affect* C. albicans *morphology switching ability, which is associated with its virulence.

**Figure 5:**
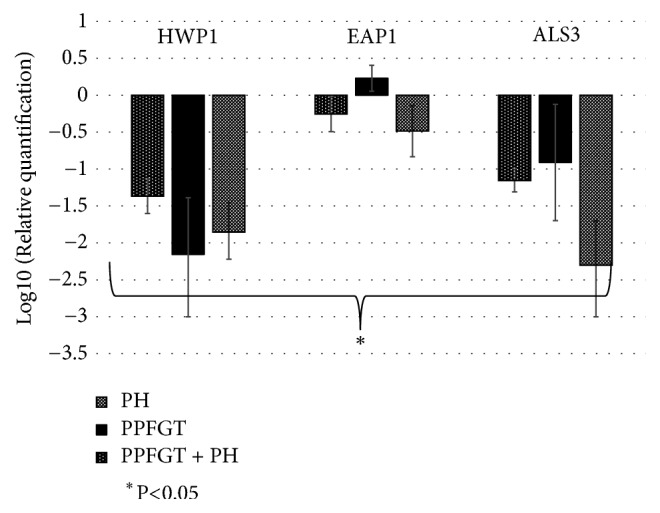
**Relative expression of selected genes.** Relative expression of the virulence associated genes (logarithmic scale), based on three independent experiments; error bars indicate standard deviation.

The gene expression results (Figure 5) indicate that PPFGT, PH, and their combination inhibited the* als3* gene; the most significant reduction was obtained using PH. PH and the PH-PPFGT combination also inhibited* eap1*. All of the treatments inhibited* hwp1* expression, PPFGT most significantly. Surprisingly, the combination of the two polyphenols did not yield the strongest reduction in the genes expression.

## 4. Discussion

*C. albicans *biofilm infections are an escalating clinical problem. Two consequences of biofilm growth with profound clinical implications are the markedly enhanced resistance to antimicrobial agents and the protection from host defenses [30].

From a dental point of view,* C. albicans* not only produces acids which causes caries, but also has a symbiotic relationship with caries-associated bacteria such as* S. mutans*. This symbiotic relationship allows the microorganisms to create an enhanced biofilm both in vitro and in vivo. Coinfected animals displayed higher levels of infection and microbial carriage within plaque biofilms compared to animals infected with either species alone. Furthermore, coinfection synergistically enhanced biofilm virulence, leading to aggressive onset of the disease with formation of rampant carious lesions [31].

In the present study, PH and PPFGT treatment affected* C. albicans *in three important manners; it reduced the number of the cells and the quantity of the EPS and inhibited the morphologic transition associated with the fungus' pathogenic capacity.

Another important finding is the specific inhibition of the polyphenols on biofilm sessile cells but not on planktonic cells. The killing of planktonic cells by medications such as azoles may result in a selection and enrichment of drug-resistant strains. Another consideration which favors biofilm inhibition is that drugs penetrate poorly into biofilms and without treatment directed at the biofilm the response is poor and temporary [32].

Shown in Figure 1 PPFGT dose-dependently inhibited the biofilm formation of* C. albicans*. This reduction was significant using concentrations of 1.25 mg/ml and higher. The addition of 0.16 mg/ml PH rather than 0.01 mg/ml resulted in a stronger reduction in biofilm generation. Therefore, we concluded that the two polyphenols have an additive effect on the inhibition of* C. albicans* biofilm formation.

This additive effect is shown also in Figures 2 and 3, describing the CLSM results.

The use of the two polyphenols together yielded the strongest inhibition on biofilm formation and EPS secretion by 88% and 74%, respectively.

Our SEM results in Figure 4 demonstrate that the morphogenic transition from yeast cells to hyphal cells may be impaired by the use of the PH+PPFGT combination. In other studies, Farnesoic acid was found to be an autoregulator of the morphogenic transition from yeast to hyphal form, produced and secreted by* C. albicans*. This autoregulator induces the transition from yeast to hypha in low concentrations but inhibits it in higher concentrations [33]. Resveratrol, a polyphenol found in grapes, and honey flavonoids also impaired this morphological alteration [34, 35].

In Figure 5, we have shown a significant downregulation in the virulence associated genes-* als3, eap1,* and* hwp1*. In similar works, curcumin, a polyphenol from the turmeric spice, showed a significant downregulation on the* als3* and* hwp1* genes [26]. In a different study, curcumin caused a downregulation of* als3* and an upregulation of the* eap1* gene [36]. An administration of 16 *μ*g/ml Thiazolidinedione-8 also resulted in an inhibition of the* als3* and* hwp1* genes [29]. In comparison, our research shows a reduced expression of all these three genes using PH and the PH + PPFGT combination. The use of PPFGT alone resulted in an upregulation of the* eap1* gene and downregulation of* als3* and* hwp1.*

Our SEM results (Figure 4) also demonstrated the capacity of the polyphenols to inhibit biofilm growth on an orthodontic PVC surface. This finding, we believe, is critical since oral appliances increase the oral* candida* carriage rate and cause a significant population alterations [12]. To our knowledge, this is the first study showing a possible mechanism for* C. albicans* inhibition on orthodontic appliances using natural agents.

PH and PPFGT showed a combined mechanism of action for the inhibition of* C. albicans* biofilm. We believe that this research may pave the way to reveal more herbal combinations for increased efficacy for* C. albicans* biofilm inhibition. In addition, PH and PPFGT might have an inhibitory effect also on biofilms formed by other microorganisms; therefore, future research is required.

## 5. Conclusion

PH and PPFGT administration dose-dependently and synergistically inhibits* C. albicans* biofilm growth* in vitro*, its ability to secrete EPS, and the yeast to hypha morphogenic change, which is crucial for the fungus virulence. It influences the total biomass of the biofilm, the morphology of the biofilm, and caries-associated properties of the biofilm, as well as the expression of the* hwp1, eap1,* and* als3* virulence associated genes. Therefore, PPFGT and PH may take part in the fight against* C. albicans* infections and ever-growing drug resistance and in caries prevention in the general population, as well as in orthodontic patients.

## Data Availability

The data used to support the findings of this study are available from the corresponding author upon request.

## Supplementary Materials

Supplementary Materials A list of primers sequences used in this study.
