# Supplementary material for: Green Tea Polyphenols and Padma Hepaten Inhibit Candida albicans Biofilm Formation
Source: Evid Based Complement Alternat Med. 2018 Sep 30;2018:1690747. doi: 10.1155/2018/1690747 (PMC6186370; doi:10.1155/2018/1690747)
Supplement: Supplementary Materials — A list of primers sequences used in this study. [file 1690747.f1.docx]

# Supplementary Material

Table of primers used in this study.
